# Double antibody pairs sandwich-ELISA (DAPS-ELISA) detects *Acidovorax citrulli* serotypes with broad coverage

**DOI:** 10.1371/journal.pone.0237940

**Published:** 2020-08-27

**Authors:** Orawan Himananto, Kirana Yoohat, Kannawat Danwisetkanjana, Mallika Kumpoosiri, Sombat Rukpratanporn, Yada Theppawong, Sudtida Phuengwas, Manlika Makornwattana, Ratthaphol Charlermroj, Nitsara Karoonuthaisiri, Petcharat Thummabenjapone, Nuttima Kositcharoenkul, Oraprapai Gajanandana

**Affiliations:** 1 National Center for Genetic Engineering and Biotechnology (BIOTEC), National Science and Technology Development Agency (NSTDA), Thailand Science Park, Pathum Thani, Thailand; 2 Department of Entomology and Plant Pathology, Faculty of Agriculture, Khon Kaen University, Khon Kaen, Thailand; 3 Department of Agriculture, Bangkok, Thailand; National Research Council Canada, CANADA

## Abstract

*Acidovorax citrulli*, a seedborne bacterium and quarantine pest, causes the devastating bacterial fruit blotch disease in cucurbit plants. Immunological assays such as ELISA are widely used in routine field inspections for this bacterium. However, to the best of our knowledge, none of the currently available monoclonal antibodies (MAbs) can detect all common *A*. *citrulli* strains. We therefore aimed to produce a panel of MAbs and to develop an ELISA-based method capable of detecting all *A*. *citrulli* strains. We used a high-throughput bead array technique to screen and characterize *A*. *citrulli*-specific MAbs produced from hybridoma clones. The hybridoma library was simultaneously screened against five *A*. *citrulli* strains (PSA, KK9, SQA, SQB and P) and the closely related bacterium, *Delftia acidovorans*. Three MAbs exhibiting different binding patterns to *A*. *citrulli* were used to develop an ELISA-based method called “double antibody pairs sandwich ELISA” (DAPS-ELISA). DAPS-ELISA employing mixtures of MAbs was able to specifically detect all 16 *A*. *citrulli* strains tested without cross-reactivity with other bacteria. By contrast, our previously developed MAb capture-sandwich ELISA (MC-sELISA) and a commercial test kit detected only 15 and 14 of 16 strains, respectively. The sensitivity of the DAPS-ELISA ranged from 5×10^5^ to 1×10^6^ CFU/mL, while those of the MC-sELISA and the commercial test kit ranged from 5×10^4^ to 1×10^7^ CFU/mL and 5×10^4^ to 5×10^5^ CFU/mL, respectively. DAPS-ELISA thus represents an alternative method enabling rapid, accurate, and inexpensive detection of all *A*. *citrulli* strains. The method can be applied to seed testing prior to planting as well as to routine field inspections.

## Introduction

*Acidovorax citrulli* (formerly *Acidovorax avenae* subsp. *citrulli*) [1] is a seedborne bacterium that causes bacterial fruit blotch in cucurbit plants, resulting in devastating outbreaks with near-total losses [2]. Outbreaks of *A*. *citrulli* impose significant economic burdens in many countries worldwide [3, 4]. Because of the devastating damage caused by this bacterium, phytosanitary certification is mandatory for cucurbit seed export, and field inspection prior to harvest and/or seed testing is required in several countries [4–7]. Efficient, reliable, and sensitive diagnostic tools for detecting *A*. *citrulli* strains are critically important for disease management. Current methods to detect *A*. *citrulli* in plant samples (leaf, rind and fruit) include bacterial isolation on selective media [8, 9], carbon source utilization profiling [10], PCR-based techniques (e.g., classical PCR, dye-based quantitative PCR [11], ethidium monoazide-PCR [12], propidium monoazide-PCR) [5], real-time PCR [13], and visual loop-mediated isothermal amplification [14]), matrix-assisted laser desorption/ionization time-of-flight mass spectrometry, Fourier transform infrared spectroscopy [15, 16], surface plasmon resonance [17], and serological assays (e.g., monoclonal antibody (MAb) capture sandwich ELISA (MC-sELISA) [18], microsphere immunoassays [19] and lateral flow immunochromatographic strips [20, 21]). ELISA-based assays are the most widely used for routine field inspection because of their simplicity, relative cost effectiveness, robustness and few requirements in terms of sample preparation, pathogen isolation or expensive equipment [22].

The diagnostic performance of ELISA-based assays, including their specificity and sensitivity, depends heavily on the characteristics of antibodies used in them. Therefore, highly specific antibodies are critical for the success of such assays. A number of polyclonal antibodies (PAbs) and MAbs directed against *A*. *citrulli* have been described [18, 20, 23] and some have been commercialized. However, to the best of our knowledge, none of the available PAbs or MAbs can detect all common *A*. *citrulli* strains. For instance, anti-*A*. *citrulli* PAbs did not react with eight *A*. *citrulli* strains (KTB 005, KTB 016, KTB 017, KTB 029, KTB 102, KTB 134, KTB 196, KTB 209) isolated from gourd, squash, and watermelon [24], while *anti-A*. *citrulli* MAbs did not react with 15 *A*. *citrulli* strains (KBT005, KBT016, KBT017, KBT029, KBT132, KBT134, KBT137, KBT148, KBT152, KBT156, KBT196, KBT209, KBT213, KBT229 and SQ B) isolated from gourd, squash and watermelon [18, 24]. One potential explanation for the inability of available antibodies to detect all *A*. *citrulli* strains is high inter-strain *A*. *citrulli* diversity. Using pathogenicity assays, DNA fingerprinting profiles, carbon utilization profiles, whole-cell fatty-acid analyses, and their type III secretion effectors, *A*. *citrulli* strains can generally be divided into at least two groups (Groups I and II) [25–27]. Group I strains have moderate to high virulence in melons and other non-watermelon cucurbits, while Group II strains are highly virulent in watermelon but weakly virulent in other cucurbits [25]. Subsequently, a third *A*. *citrulli* group comprising two strains (ZUM4000 and ZUM4001) was identified by Eckshtain-Levi et al. (2014). This additional group was weakly virulent in watermelon, melon, and squash seedlings [27].

Because of the high diversity of *A*. *citrulli* strains, MAbs and diagnostic tests capable of detecting all strains of *A*. *citrulli* are urgently needed. Therefore, we aimed to produce MAbs specific to *A*. *citrulli* using membrane protein extracts of *A*. *citrulli* as immunogens. A high-throughput bead array technique was developed to screen for *A*. *citrulli*-specific antibodies produced from hybridoma clones. This bead array enabled multiplex screening against five *A*. *citrulli* strains as well as a closely related bacterium in the family *Comamonadaceae*, *Delftia acidovorans* [18, 23]. Specific reactive MAbs were selected and used to develop an efficient ELISA capable of detecting all *A*. *citrulli* strains circulating in Thailand.

## Materials and methods

### Bacterial strains

Information on all bacterial strains used in this study are provided in Table 1. *A*. *citrulli* strains were identified by pathogenicity assays on various cucurbit seedlings and confirmed by PCR using primers specific for the 16S rRNA gene [28, 29]. Bacteria were cultured on tryptic soy agar with yeast extract (M011, Himedia, India) at 30°C for 48 h. A single colony was used to inoculate 50 mL of tryptic soy broth with yeast extract, and the culture was grown at 30°C for 18 h with 200 rpm shaking. Growth of bacterial cultures was assessed by measuring the optical density at 600 nm (OD600) using a spectrophotometer (Spectro UV-VIS RS; Labomed Inc., Los Angeles, CA). An OD600 of 1.0 was equivalent to 1×10^9^ colony-forming units (CFU)/mL [18]. Bacterial cells were harvested by centrifugation at 5,000 × *g* for 10 min (RC 5C Plus, Thermo Fisher, Waltham, MA). The cell pellets were washed twice with 5 mL of phosphate-buffered saline (PBS) and resuspended in 5 mL of PBS to obtain a final concentration of 1×10^10^ CFU/mL.

**Table 1:** Bacterial strains used in this study.

Bacterial strains	Host	Location	Source^a^	Year
*Acidovorax citrulli* (Ac)				
Ac AA	Watermelon	Mahasarakham, Thailand	DPAK	2009
Ac BB	Squash	Imported seeds^b^	DPAK	2009
Ac K	Squash	Imported seeds^b^	DPAK	2009
Ac KK9	Watermelon	Khon Kaen, Thailand	DPAK	1993
Ac KKU	Watermelon	Khon Kaen, Thailand	DPAK	2018
Ac M1	Melon	Chiang Mai, Thailand	AGRI CMU	2018
Ac M2	Melon	Chiang Mai, Thailand	AGRI CMU	2018
Ac P	Squash	Imported seeds^b^	DPAK	2009
Ac P1	Melon	Lampoon, Thailand	AGRI CMU	2018
Ac P2	Melon	Lampoon, Thailand	AGRI CMU	2018
Ac PSA	Watermelon	Nakhon Ratchasima	DOA	1995
Ac SQ A	Squash	Imported seeds^b^	DPAK	2009
Ac SQ B	Squash	Imported seeds^b^	DPAK	2010
Ac Q	Bottle gourd	Imported seeds^b^	DPAK	2009
Ac WM 001	Watermelon	Khon Kaen, Thailand	DPAK	1993
Ac Z	Bottle gourd	Imported seeds^b^	DPAK	2009
*A*. *cattleyae*	Orchid	Ratchaburi, Thailand	DOA	2006
*A*. *avenae*	Corn	NA^c^	DPAN	NA^c^
*Acinetobacter* spp.	Watermelon	Khon Kaen, Thailand	BIOTEC	2018
*Delftia acidovorans*	Watermelon	NA^c^	DPAK	2009
*Pantoea ananus*	Tomato	NA^c^	DPAK	2019
*Pectobacterium carotovorum*	Chinese Cabbage	NA^c^	DPAK	2019
*Pseudomonas* spp.	Cucurbits	NA^c^	DPAK	2019
*Stenotrophomonas* spp.	Cucurbits	Khon Kaen, Thailand	BIOTEC	2018

^a^ Sources from Thailand: AGRI CMU, Faculty of Agriculture, Chiang Mai University, Chiang Mai; DOA, Department of Agriculture, Ministry of Agriculture and Cooperatives; DPAN, Department of Plant Pathology, Kasetsart University, Kamphaeng Saen Campus, Nakhon Pathom; BIOTEC, National Center for Genetic Engineering Biotechnology; DPAK, Department of Plant Sciences and Agricultural Resources, Khon Kaen University, Khon Kaen.

^b^ Isolates were obtained during seedling grow-out tests for bacterial fruit blotch pathogens. All tested seedlings were sterilized by autoclaving following examination.

^c^ NA: Information not available.

### Antigen preparation

The membrane protein complex (MPC) of *A*. *citrulli* strain PSA was used as the antigen for MAb production. The MPC was prepared using a previously published protocol with some modifications [30]. Briefly, the bacterial pellet was resuspended in 40 mL of 0.2 M LiCl. Glass beads (3 mm in diameter, 60 g glass beads) were used to break the suspended cells by shaking at 200 rpm at 45°C for 3 h and removed by filtration through two layers of gauze. Bacterial cell debris was removed by centrifugation at 15,000 × *g*, 4°C for 1 h. The supernatant containing MPC was further concentrated by centrifugation at 100,000 × g, 4°C for 2 h and stored at -20°C.

### MAb production

The protocol for MAb production using the hybridoma method was modified from Oi and Herzenberg [31]. Six-week-old BALB/c mice were injected intraperitoneally (i.p.) with 100 μg of Ac PSA MPC. Three immunizations were performed at 2-week intervals. Serum from each immunized mouse was obtained 7 days after each immunization and tested for antibodies against an *A*. *citrulli* cell suspension using the bead array method described below. The mouse with the highest antibody titer was boosted i.p. with 120 μg of the antigen and sacrificed 3 days later for hybridoma preparation. Splenocytes were fused with P3X63-Ag8.653 murine myeloma cells (splenocyte:myeloma cell ratio 5:1) in the presence of 50% (w/v) polyethylene glycol (P7181, Sigma, Munich, Germany). Hybridoma cells were selected by culturing in hypoxanthine aminopterin thymidine (HAT) medium, consisting of RPMI 1640 medium (Invitrogen, Carlsbad, CA), 20% (v/v) fetal bovine serum (PAA Laboratories, Pasching, Austria), 1× HAT supplement (H0262, Sigma), and 10% (v/v) BM-Condimed H1 (Hybridoma Cloning Supplement, Roche, Basel, Switzerland). Hybridoma cultures were propagated and screened for production of antibodies against *A*. *citrulli* using a bead array method. The animal study protocol was approved by the Institutional Animal Care and Use Committee of the National Center for Genetic Engineering and Biotechnology (Protocol Number: BT-Animal 03/2561) and was carried out in strict accordance with the recommendations of the Ethical Principles and Guidelines for the Use of Animals from the National Research Council of Thailand. All efforts were made to minimize animal suffering.

### Bead array for hybridoma screening

Six MPCs (Ac KK9, Ac SQ A, Ac SQ B, Ac P, Ac PSA, and *D*. *acidovorans*) were used as antigens for hybridoma screening. Each antigen was linked to a fluorescently barcoded microsphere according to the manufacturer’s instructions (Luminex, Austin, TX). Empty microspheres without any antigens were used to evaluate non-specific binding. Briefly, 1×10^5^ microspheres were washed twice with activation buffer (100 mM NaH_2_PO_4_, pH 6.2) and then activated with sulfo-N-hydroxysulfosuccinimide (50 mg/mL, #24510, Thermo Scientific) and 1-ethyl-3-[3-dimethylaminopropyl] carbodiimide hydrochloride (50 mg/mL, #22980, Thermo Scientific) for 20 min at room temperature (RT) with shaking. The activated microspheres were washed three times using a magnetic tube separator. Antigen (5 μg of each MPC) was added and incubated in the dark with shaking for 2 h at RT. Excess uncoupled antigen was removed by washing with PBST (PBS, pH 7.4, containing 0.05% (v/v) Tween 20) using a magnetic tube separator. Conjugated microspheres were stored at 4°C until use.

To perform hybridoma screening, a mixture of the seven microsphere sets (10^3^ microspheres per set in a total volume of 50 μL of PBST containing 2% (w/v) bovine serum albumin (BSA; Sigma, #5890)) was added into each well of a 96-well microplate (#650101; Greiner, Kremsmünster, Austria). Hybridoma culture supernatant (50 μL) was added and incubated for 1 h at RT in the dark with shaking. The microspheres were washed three times with 100 μL of PBST using a Bio-Plex Pro wash station (Bio-Rad, Hercules, CA). R-phycoerythrin (RPE)-labeled anti-mouse IgG (100 μL/well, 2 μg/mL, #P852, Invitrogen) was added into each well and incubated for 1 h in the dark with shaking. A wash step was performed to remove unbound RPE-labeled antibodies. The microspheres were resuspended in 100 μL of PBST and detected using a MAGPIX^®^ detector (Luminex). For each sample, the median fluorescent intensity (MFI) values for all microsphere sets were recorded. MAb 11E5 [18] and anti-*A*. *citrulli* serum (PAb) from an immunized mouse were used as positive controls. RPMI medium was used as a negative control. A signal at least three times that of the negative control was considered positive. Single hybridoma cells producing antibodies specific for *A*. *citrulli* were obtained by the limiting dilution method.

### Plate trapped antigen-ELISA (PTA-ELISA)

The PTA-ELISA was performed by resuspending MPCs in a sodium bicarbonate buffer. The suspensions (100 μL/sample) were added into each well of a 96-well microplate and incubated overnight at 4°C. The plate was washed with PBST and blocked with 2% BSA in PBST for 1 h at RT. Wells were washed with PBST and hybridoma culture medium was added to each well and incubated for 1 h at RT. The wells were washed again with PBST and an alkaline phosphatase (AP)-conjugated goat anti-mouse secondary antibody was added and incubated for 1 h at RT. After a final wash with PBTS, *p*-nitrophenyl phosphate (pNPP, 002201, Invitrogen) substrate was added and incubated for 30 min. The absorbance at 405 nm was measured using a spectrophotometer (SpectraMax^®^ M5, San Jose, CA). Each sample was assayed in triplicate and the mean signal was reported. RPMI medium was used as a negative control. A signal at least twice that of the negative control signal was considered positive [32].

### Development of DAPS-ELISA

The double antibody pairs sandwich-enzyme linked immunosorbent assay (DAPS-ELISA) was modified from a previously reported MC-sELISA protocol [18]. Various combinations of MAbs (11E5, 7G9 and 14B6) were used as capture and detection antibodies. Briefly, MAb mixtures (2 μg/mL in sodium bicarbonate, pH 9.6, 100 μL) were coated in each well of a 96-well microplate overnight at 4°C. Bacterial suspensions in healthy leaf sap (100 μL/well) were added to the wells and incubated for 1 h at RT. The wells were washed with PBST and then a mixture of AP conjugated MAbs was added and incubated for 1 h at RT. Each well was washed with PBST and pNPP substrate was added. Absorbance was measured at 405 nm using a spectrophotometer. Each sample was measured at least twice and mean signals were reported. Healthy plant sap with no bacterial cells was used as a negative control. A signal at least twice that of the negative control was considered positive.

### Comparison of the specificity and sensitivity of ELISA systems for detection of *A*. *citrulli* in plant sap

Three sandwich ELISA systems (DAPS-ELISA, MC-sELISA, and a commercial ELISA kit) for detection of *A*. *citrulli* were compared. DAPS-ELISA using two MAb pairs (MAbs 11E5+14B6 as capture antibodies and MAbs 11E5+7G9 as detection antibodies) was performed as described above. MC-sELISA was performed according to our previously published protocol [18]. Briefly, a MAb (11E5) specific to *A*. *citrulli* (2 μg/mL in sodium bicarbonate, pH 9.6, 100 μL) was coated in each well of a 96-well microplate overnight at 4°C. Bacterial suspensions (20% w/v) in healthy plant sap (pulverized watermelon leaves extracted in PBST containing 2% ovalbumin, 10 mM sodium sulfite, and 0.5 mM polyvinylpyrrolidone) were added to wells (100 μL/well) and incubated for 1 h at RT. The wells were washed with PBST and a PAb specific to *A*. *citrulli* (BIOTEC, NSTDA) was added and incubated for 1 h at RT. Each plate was washed again with PBST and an AP conjugated anti-rabbit antibody (A3937, Sigma) was added and incubated for 1 h at RT. After a final wash with PBST, pNPP substrate was added. The absorbance at 405 nm was measured using a spectrophotometer. Each sample was assayed in triplicate and the mean signal was reported. Healthy plant sap sample (without bacterial cells) was used as a negative control. A signal at least twice that of the negative control was considered positive.

The commercial ELISA detection kit for *A*. *citrulli* was purchased from Agdia Company (#SRA27200, Elkhart, IN) and used according to the manufacturer’s instructions.

## Results

### Production and screening of MAbs

**Fig 1:**
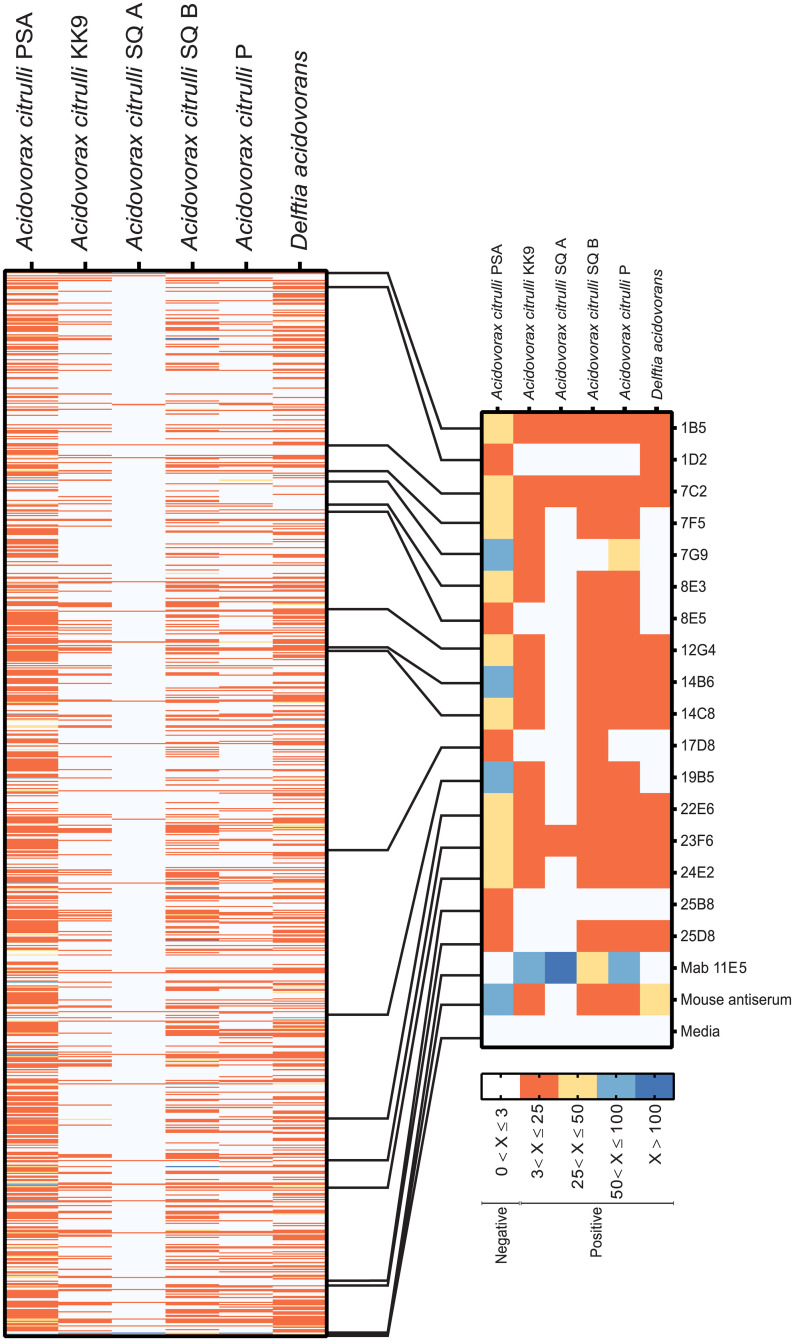
A hybridoma library was screened against MPCs of five *A*. *citrulli* (Ac) strains and *Delftia acidovorans* using a high-throughput bead array method. Each row represents a hybridoma clone and each column represents the signals obtained using the indicated antigen. The right inset shows the selected 17 culture supernatants reacted strongly with Ac PSA-linked microspheres (MFI > 300) with signals more than three-fold higher than those against *D*. *acidovorans*-linked microspheres. Mouse MAb 11E5 [18] and mouse antiserum were used as positive controls. RPMI media was used as a negative control. The scale bar indicates signal-to-background ratios; ratios ≤ 3 were considered negative.

To produce MAbs specific to *A*. *citrulli*, a hybridoma library was generated using the MPC of Ac PSA. A high-throughput bead array method was used to screen for hybridoma clones producing *A*. *citrulli*-specific antibodies. Culture supernatants were simultaneously screened against fluorescently barcoded microbeads coupled to MPCs of five different *A*. *citrulli* strains and *D*. *acidovorans*. Of 1500 fusion wells, 830 contained viable hybridoma cells (55%) and were tested using the bead array method. Among these 830 hybridoma supernatants, 202 reacted with Ac PSA with different patterns of reactivity against other Ac strains. Seventeen culture supernatants reacted strongly with Ac PSA-linked microspheres (MFI > 300) with signals more than three-fold higher than those against *D*. *acidovorans*-linked microspheres were selected for cloning by limiting dilution (Fig 1). After three successive rounds of subcloning, two stable hybridoma cell lines secreting MAbs 7G9 and 14B6 were identified and selected for further characterization. The other 15 hybridoma lines were unstable and lost their antibody productivity. MAb 11E5, a positive control obtained in our previous study [18], is known to react with four *A*. *citrulli* strains but does not recognize Ac PSA or *D*. *acidovorans*. Mouse serum, another positive control, reacted with all bacteria tested except *A*. *citrulli* strain SQA. RPMI media, a negative control, did not bind to any bacteria.

### Characterization of MAbs

**Fig 2:**
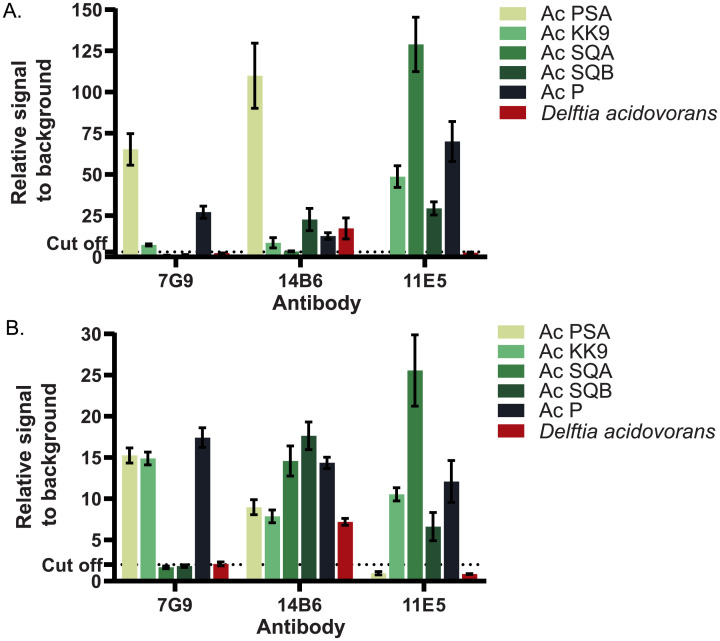
Comparison of reactivity patterns of three MAbs against five different *A*. *citrulli* strains and *Delftia acidovorans* using a bead array and PTA-ELISA. Binding of MAbs 7G9, 14B6 and 11E5 was tested against five different *A*. *citrulli* strains (PSA, KK9, SQA, SQB and P) and *Delftia acidovorans* using a bead array (A) and PTA-ELISA (B). Each dataset was plotted as an average of triplicate measurements with an error bar indicating the standard deviation. The dotted line represents three-fold the signal of the negative control (coating buffer).

The reactivity of MAbs 7G9 and 14B6 was further evaluated against five *A*. *citrulli* strains (PSA, KK9, SQA, SQB and P) and *D*. *acidovorans*, a closely related bacterium in the family Comamonadaceae, using the bead array and PTA-ELISA (Fig 2). MAb 7G9 (isotype IgG1, kappa) reacted with three strains of *A*. *citrulli* (PSA, KK9 and P) and did not cross-react with *D*. *acidovorans*. By contrast, MAb 14B6 (isotype IgG1, kappa) reacted with all five *A*. *citrulli* strains tested as well as *D*. *acidovorans* (Fig 2). The results of bead array and PTA-ELISA revealed similar reactivity patterns for each MAb and each bacterial antigen (Fig 2). MAb 11E5 [18] reacted with four *A*. *citrulli* strains tested (KK9, SQA, SQB and P) and did not cross-react with *D*. *acidovorans* (Fig 2). The commercial MAbs (Agdia) reacted to all tested strains except SQB and P (S1 Table).

### DAPS-ELISA for detection of *A*. *citrulli*

**Fig 3:**
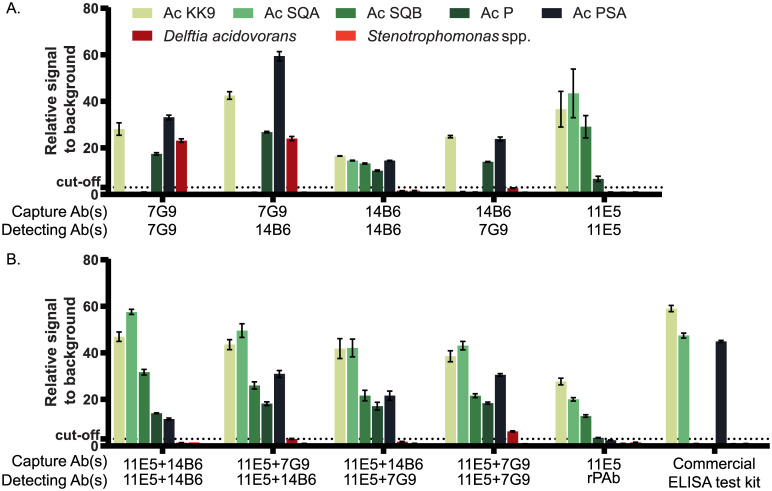
Development of DAPS-ELISA for detection of *A*. *citrulli* using three monoclonal antibodies (7G9, 14B6 and 11E5). Sandwich ELISA using (A) single antibodies and (B) mixtures of two monoclonal antibodies as capture and detection antibodies. A commercial ELISA kit (Agdia) was used to compare the spectrum of *A*. *citrulli* strains detected. Each dataset was plotted as an average of triplicate measurements with an error bar indicating the standard deviation. The dotted line represents three-fold the signal of the negative control (healthy plant sap).

Three MAbs (7G9, 14B6 and 11E5) were used to develop a sandwich ELISA for specific detection of all circulating *A*. *citrulli* strains in plant samples. To select antibody pairs for broad detection of *A*. *citrulli* strains, all possible combinations of these three MAbs were evaluated. When a single antibody was used as a capture and detection antibody in ELISA (MAbs 7G9/7G9, 7G9/14B6, 14B6/7G9, or 11E5/11E5), the resulting assays were not able to detect all strains of *A*. *citrulli* (KK9, SQA, SQB, P and PSA) (Fig 3A). For example, ELISA using MAb 7G9 as a capture antibody in combination with either MAb 7G9 or MAb 14B6 as a detection antibody was unable to detect *A*. *citrulli* strains SQA and SQB and showed cross-reactive detection of *D*. *acidovorans* (Fig 3A). Some ELISA formats (MAbs 14B6/7G9 and 11E5/11E5) were unable to detect all *A*. *citrulli* strains tested but did not cross-react with other bacteria. ELISA using MAb 14B6 as a capture antibody and MAb 7G9 as a detection antibody was unable to detect *A*. *citrulli* strains SQA and SQB, while the ELISA format using MAb 11E5 as both the capture and detection antibody could not detect *A*. *citrulli* strain PSA. Some ELISA formats (e.g., MAb 14B6/14B6) could detect all *A*. *citrulli* strains tested but with extremely low signals. All tests were performed in parallel with MC-sELISA (MAb 11E5/rPAb) [18] and a commercial ELISA kit (Agdia). MC-sELISA was unable to detect *A*. *citrulli* strain PSA while the commercial ELISA kit could not detect *A*. *citrulli* strains SQB and P.

In contrast to ELISA formats using a single antibody, when MAb 11E5 was combined with either MAb 7G9 or MAb 14B6 as capture and detection antibodies, three combinations (MAbs 11E5+14B6/11E5+14B6, 11E5+7G9/11E5+14B6, 11E5+14B6/11E5+7G9) could detect all *A*. *citrulli* strains tested with no cross-reactivity against other bacteria (Fig 3B). Given its high reactivity with all *A*. *citrulli* strains tested and low reactivity against other bacteria, the system employing MAbs 11E5 and 14B6 as capture antibodies and MAbs 11E5 and 7G9 as detection antibodies was selected for further characterization.

### Characterization of DAPS-ELISA

**Fig 4:**
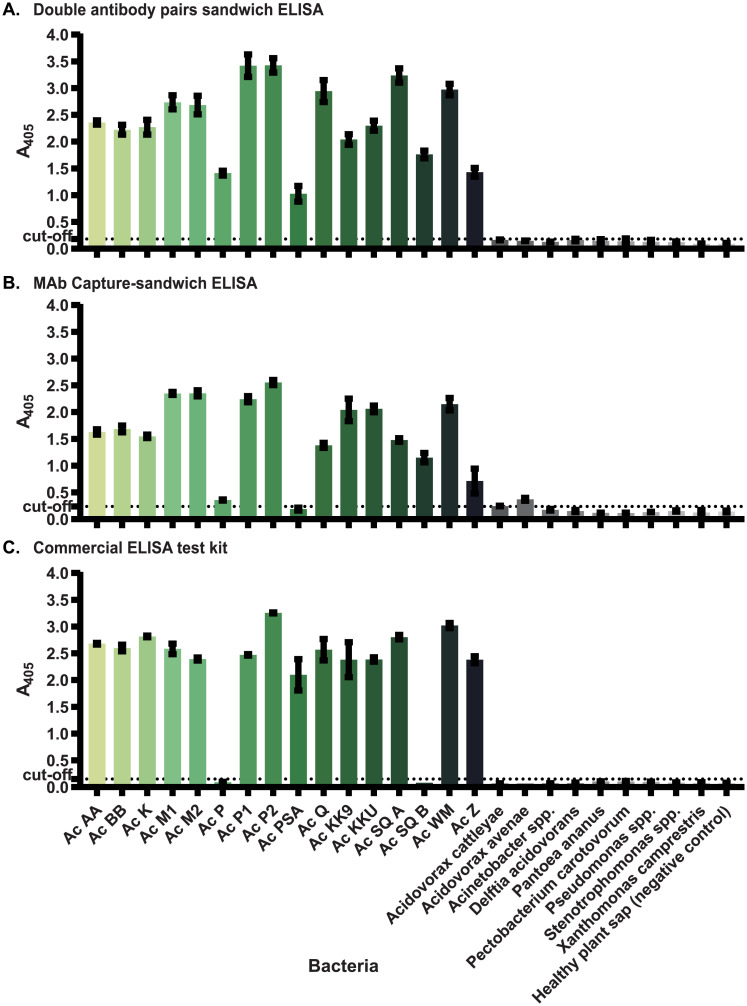
Comparison of the specificities of three sandwich ELISA methods for detection of *Acidovorax citrulli*. (A) DAPS-ELISA using three monoclonal antibodies (11E5, 7G9 and 14B6), (B) MC-sELISA and (C) a commercial ELISA test kit (Agdia) were used to detect the indicated bacteria. Each dataset was plotted as an average of triplicate measurements with an error bar indicating the standard deviation. The dotted line represents three-fold the signal of the negative control (coating buffer).

The specificities of three sandwich ELISA methods (the newly established DAPS-ELISA using two MAb pairs, MC-sELISA [18], and a commercial ELISA test kit (Agdia)) were compared for detection of various *A*. *citrulli* strains and other bacteria (Fig 4). DAPS-ELISA detected all 16 *A*. *citrulli* strains tested and did not cross-react with other bacteria. MC-sELISA detected almost all *A*. *citrulli* strains tested (15 of 16) but did not detect Ac PSA and cross-reacted slightly with *A*. *avenae*. The commercial test kit (Agdia) detected 14 of 16 *A*. *citrulli* strains tested. This commercial test kit did not detect Ac SQB and P and did not cross-react with other bacteria.

To evaluate the sensitivities of the three sandwich ELISA methods, each of the five *A*. *citrulli* strains were serially diluted in healthy plant sap and subjected to each ELISA method. The DAPS-ELISA detected all five *A*. *citrulli* strains tested with detection limits ranging from 5×10^5^ to 1×10^6^ CFU/mL (Table 2). MC-sELISA detected 4 of 5 *A*. *citrulli* strains with detection limits ranging from 5×10^4^ to 1×10^7^ CFU/mL. The commercial test kit detected only 3 of 5 *A*. *citrulli* strains with detection limits ranging from 5×10^4^ to 5×10^5^ CFU/mL (Table 2).

**Table 2:** Detection limits of DAPS-ELISA, MC-sELISA and the commercial test kit for detection of *Acidovorax citrulli* strains.

*Acidovorax citrulli*	DAPS-ELISA(CFU mL^-1^)	MC-sELISA(CFU mL^-1^)	Commercial kit(CFU mL^-1^)
Ac KK9	5×10^5^	5×10^4^	5×10^4^
Ac SQA	5×10^5^	5×10^4^	5×10^4^
Ac SQB	1×10^6^	1×10^6^	ND
Ac P	1×10^6^	1×10^7^	ND
Ac PSA	1×10^6^	ND	5×10^5^

ND = Not detectable.

## Discussion

Accurate diagnostic tests that can specifically detect all strains of *A*. *citrulli* are essential for disease management and phytosanitary certification. To successfully develop diagnostic tests capable of detecting all *A*. *citrulli* strains based on immunoassay principles, it is crucial to obtain antibodies that can strongly and specifically react with all strains. However, currently available antibodies show either cross-reactivity with closely-related bacteria or inability to detect all *A*. *citrulli* strains [23, 24]. For instance, Walcott *et al*. [23] produced a PAb that reacted with all *A*. *citrulli* strains tested but cross-reacted with *A*. *avenae* (formerly *A*. *avenae* subsp. *avenae*), *A*. *cattleyae* (formerly *A*. *avenae* subsp. *cattleyae*), and the closely related bacterium *D*. *acidovorans* (formerly *Comomonas acidovorans*) [1]. A commercial MAb (Agdia) specifically reacted with *A*. *citrulli* but was unable to detect some strains of *A*. *citrulli* isolated from watermelon, squash and ornamental gourd [18, 24]. A specific MAb against *A*. *citrulli* (MAb 11E5) was generated by our team [18]. However, after collecting a large number of *A*. *citrulli* strains from cucurbit production fields in Thailand and from imported seeds, we found that this MAb could not detect an *A*. *citrulli* strain isolated from watermelon (Ac PSA). Therefore, the aims of this study were to produce MAbs specific to *A*. *citrulli* strain PSA and to use these MAbs in the development of a new ELISA method that can specifically detect all available *A*. *citrulli* strains.

In order to produce highly specific MAbs capable of recognizing all *A*. *citrulli* strains, both the type of immunogen and high-throughput hybridoma screening methods were considered in this study. The MPC of Ac PSA was used as the immunogen for MAb production because it was reported to be more immunogenic than other components and is potentially conserved in all groups within the genus [33, 34]

Hybridoma screening is one of the most important steps in MAb production. Thousands of individual hybridoma cultures must be tested at the same time for desired antibody properties. A conventional screening method such as ELISA is time consuming and laborious. Moreover, ELISA can screen each hybridoma culture against only 2–3 antigens at once due to volume limitations of hybridoma supernatants in the original fusion wells (~150–200 μL/well). When a hybridoma library is screened against multiple antigens, expansion of hybidoma cultures is required, increasing time and workload. Therefore, we developed a high-throughput bead array technique to enable multiplex screening of hybridoma clones against five *A*. *citrulli* strains and *D*. *acidovorans* simultaneously in one well. The bead array technique presents an alternative method with the potential to screen and characterize hybridoma cultures against up to 50 antigens per sample, allowing rapid determination of cross-reactivity against a panel of the antigens of interest. The results of high-throughput hybridoma screening facilitate prompt clone selection for subsequent cloning, accelerating successful production of MAbs with desired properties [35–37].

Following immunization with *A*. *citrulli* strain PSA MPC and bead array screening of hybridoma supernatants, we were able to select stable hybridoma lines secreting two MAbs, 7G9 and 14B6, with different properties. Hybridoma screening by bead array and PTA-ELISA revealed similar reactivity patterns for each MAb and each bacterial antigen. MAb 7G9 reacted with three *A*. *citrulli* strains (PSA, KK9 and P) and did not cross-react with *D*. *acidovorans*, while MAb 14B6 reacted with all five *A*. *citrulli* strains tested (PSA, KK9, SQA, SQB and P) as well as *D*. *acidovorans*. The characteristics of both MAbs produced in this study differ from those of MAb 11E5, previously produced by our team against a sonicated cell suspension of *A*. *citrulli* strain KK9, which reacted with four *A*. *citrulli* strains tested (KK9, SQA, SQB and P) and did not cross-react with *D*. *acidovorans* [18].

Given their different specificities, each MAb alone was unable to detect all strains of *A*. *citrulli*. However, combining these MAbs enabled development of a superior sandwich ELISA method for detection of *A*. *citrulli*. The DAPS-ELISA employing MAb 11E5 in combination with MAb 14B6 as capture antibodies and MAb 11E5 in combination with MAb 7G9 as detection antibodies was able to detect all 16 *A*. *citrulli* strains tested without cross-reactivity with other bacteria. The specificity of the newly developed DAPS-ELISA was superior to that of MC-sELISA using MAb 11E5 as capture antibody and anti-Ac MPC PAb as detection antibody [18] and that of the commercial test kit (Agdia); both of the latter two assays were unable to detect some strains of *A*. *citrulli*. However, for detecting some *A*. *citrulli* strains, the DAPS-ELISA was less sensitive than the MC-sELISA [18] and Agdia test kit. For instance, the sensitivity of DAPS-ELISA in detection of three *A*. *citrulli* strains (AC KK9, Ac SQA and Ac PSA) was ten times lower than those of the MC-sELISA and /or Agdia test kit. Nevertheless, DAPS-ELISA and MC-sELISA could detect Ac SQB with the same sensitivity, while Agdia test kit could not detect this strain at all. Moreover, DAPS-ELISA was ten times more sensitive than MC-sELISA in detection Ac P, while Agdia test kit could not detect this strain. In order to improve detection sensitivity in some *A*. *citrulli* strains, signal enhancing techniques could be further applied [38, 39].

Generally, *A*. *citrulli* strains can be divided into at least two groups (Groups I and II) based on pathogenicity assays, DNA fingerprinting profiles, and whole-cell fatty-acid analyses [25, 26]. Group I mainly includes strains exhibit a moderate to high level of virulence on melon and other non-watermelon cucurbits, while Group II highly virulent on watermelon but weakly virulent on other cucurbits [25–27]. All 16 *A*. *citrulli* strains used in this study have been characterized by 16S rRNA sequencing and pathogenicity assays on various cucurbit hosts (unpublished data). Based on pathogenicity tests, four strains (Ac PSA, Ac SQA, Ac SQB and Ac P) are classified in Group I, while the remaining 12 strains (Ac AA, Ac BB, Ac K, Ac KK9, Ac KKU, Ac M1, Ac M2, Ac P, Ac P1, Ac P2, Ac Q, Ac WM 001 and Ac Z) are classified in Group II. To characterize our MAbs and develop the DAPS-ELISA, five *A*. *citrulli* strains (Ac KK9, Ac P, Ac PSA, Ac SQA and Ac SQB) were selected as representatives of both groups. While DAPS-ELISA was able to detect both groups achieving the goals of the study, it was unable to distinguish between the two groups.

In conclusion, the MAbs obtained in this study from a hybridoma library using a high-throughput bead array technique can be used in a DAPS-ELISA that can specifically detect all *A*. *citrulli* strains tested. This newly developed ELISA presents an alternative method for rapid, sensitive, accurate, and inexpensive detection of *A*. *citrulli* with broad strain coverage. The method can be applied to seed testing prior to planting and to routine field inspections. However, the DAPS-ELISA requires further validation in other laboratories to ensure its reliability.

## Supporting information

S1 Table Binding activities of selected bacteria for characterization against panel of antibodies. (DOCX)
