# Supplementary material for: Double antibody pairs sandwich-ELISA (DAPS-ELISA) detects Acidovorax citrulli serotypes with broad coverage
Source: PLoS One. 2020 Aug 27;15(8):e0237940. doi: 10.1371/journal.pone.0237940 (PMC7451559; doi:10.1371/journal.pone.0237940)
Supplement: S1 Table — (DOCX) [file pone.0237940.s001.docx]

**Supplementary Table S1.**  Binding activities of selected bacteria for characterization against panel of antibodies

| Bacterial strains | Group  (Based on pathogenicity) | Previously available MAbs | | | Newly developed MAbs | |
| --- | --- | --- | --- | --- | --- | --- |
|  |  | MAb 11E5 | Agdia capture MAb | Agdia detection MAb | MAb 7G9 | MAb 14B6 |
| *Acidovorax citrulli* (Ac) |  | | | | | |
| Ac KK9 | II | + | + | + | + | + |
| Ac P | I | + | - | - | + | + |
| Ac PSA | I | - | + | + | + | + |
| Ac SQ A | I | + | + | + | - | + |
| Ac SQ B | I | + | - | - | - | + |
| *Delftia acidovorans* |  | - | - | - | - | + |

"+" indicate a positive signal from the designated monoclonal antibody and the bacterial strain.

"-" indicate a negative signal from the designated monoclonal antibody and the bacterial strain.
